# Rotavirus Genotype Distribution after Vaccine Introduction, Rio de Janeiro, Brazil

**DOI:** 10.3201/eid1501.071136

**Published:** 2009-01

**Authors:** Filipe Anibal Carvalho-Costa, Irene Trigueiros Araújo, Rosane Maria Santos de Assis, Alexandre Madi Fialho, Carolina Maria Miranda de Assis Martins, Márcio Neves Bóia, José Paulo Gagliardi Leite

**Affiliations:** Oswaldo Cruz Institute, Rio de Janeiro, Brazil (F.A. Carvalho-Costa, I.T. Araujo, R.M. Santos de Assis, A.M. Fialho, M.N. Bóia, J.P.G. Leite); Salles Netto Municipal Hospital, Rio de Janeiro, Brazil (C.M.M. de Assis Martins); Rio de Janeiro State University, Rio de Janeiro, Brazil (M.N. Bóia)

**Keywords:** Rotavirus, vaccine, genotypes, Brazil, dispatch

## Abstract

Brazil introduced rotavirus vaccination in March 2006. We studied 133 rotavirus-positive fecal samples collected from February 2005 through December 2007. Genotype G2P[4] was found in 1.4% of samples in 2005, in 44% in 2006, and in 96% in 2007. Rotavirus detection rate decreased from 38% in 2005 to 24% in 2007 (p = 0.012).

Group A rotaviruses (RV-A) are the major etiologic agents of acute diarrhea in infants, causing ≈611,000 deaths each year (*1*). The recently developed attenuated G1P[8] vaccine, Rotarix (GlaxoSmithKline, Rixensart, Belgium), was included in the Brazilian Expanded Immunization Program and, after March 2006, became available to the whole birth cohort. Rio de Janeiro is the second largest Brazilian city; vaccine coverage was 43.3% in 2006 and 74.4% in 2007. Although Rotarix was highly efficacious for preventing severe RV gastroenteritis in phase III trials carried out in Latin America and Europe, it appears to be less effective in preventing diarrhea caused by G2P[4] RV-A strains, which do not share either the VP7 or the VP4 surface antigen with the vaccine strain (*2*).

Initial studies carried out in northeastern Brazil after RV-A vaccine introduction demonstrated the predominance of RV-A G2P[4] in vaccinated populations (*3**–**5*). Also, the apparent extinction of non-G2 rotavirus strains from circulation was associated with a significant reduction in the frequency of RV-A detection in children with gastroenteritis (*5*). This finding suggests that G2P[4] strains could be, to some extent, replacing P[8] genotypes in the postvaccination period.

Sentinel RV-A surveillance, performed in selected pediatric settings as part of the strategies of immunization programs in Latin America, has been recommended to better assess RV-A effects and strain characterization. In this context, studies carried out in Brazil demonstrated that the emergence of G9P[8] RV-A in Rio de Janeiro in the late 1990s was accompanied by the disappearance of common genotypes like G2P[4] and G3P[8] and the continuous detection of RV-A genotype G1P[8] (*6*–*10*). RV-A G5, an atypical genotype prevalent in the early 1990s, has not been detected in Rio de Janeiro since 1997 (*6*–*10*). In this study, we estimated the distribution of RV-A genotypes in hospitalized children in Rio de Janeiro before and after the monovalent RV-A vaccine was introduced into the national immunization schedule**.**

## The Study

From February 2005 through December 2007, fecal samples were collected from 464 hospitalized children from birth to 5 years of age who exhibited gastroenteritis and dehydration and required intravenous fluid replacement. The study was conducted in Salles Netto Municipal Hospital, a pediatric unit in Rio de Janeiro.

Most children studied (390 [84%]) were not eligible for full vaccination; they either were born before January 1, 2006, or were <4 months of age. Nevertheless, 39 (8.4%) had been vaccinated with 2 doses of Rotarix, and 35 (7.5%) did not receive the vaccine.

Samples were collected after written consent was given by the parents. This study was approved by the Oswaldo Cruz Foundation Ethical Research Committee (protocol no. 311/06).

Polyacrylamide gel electrophoresis and a combined enzyme immunoassay for RV-A strains and adenoviruses were used to detect RV-A. Most samples were G- and P-typed through seminested reverse transcription–PCR, as described (*11*). Seventeen RV-A–positive samples were P-typed through partial genome sequencing. This method was also used to G-type 1 sample. These strains could not be typed through PCR. All samples that were P-typed through sequencing were P[8]. The only sample G-typed through sequencing was G9.

RV-A strains were detected in 133 (29%) of 464 samples. Genotype distribution showed a different profile for each year: 45% G9P[8], 30% G3P[8], 14% G1P[8], and 1.4% G2P[4] in 2005; 41% G2P[4], 18% G3P[8], and 15% G9P[8] in 2006; and 96% G2P[4] in 2007 (Table).

**Table:** Frequency of rotavirus A infection and distribution of G and P genotypes from February 2005 through December 2007, Rio de Janeiro, Brazil*

*Vaccination status and year*	*No. samples*	*No. (%) rotavirus* *positive*	*No. (%) G1P[8]*	*No. (%) G2P[4]*	*No. (%) G3P[8]*	*No. (%) G9P[8]*	*No. (%) other genotypes, mixed or not typeable*
Ineligible for full vaccination†							
2005	193	73 (38)	10 (14)	1 (1.4)	22 (30)	33 (45)	7 (9.5)
2006	148	34 (23)	1 (2.9)	14 (41)	6 (8)	5 (5)	8 (23)
2007	49	12 (24)	1 (8)	11 (92)	–	–	–
Vaccinated‡							
2006	6	Negative§					
2007	33	4 (12)	–	4 (100)	–	–	–
Not vaccinated							
2006	8	Negative§					
2007	27	10 (37)	–	10 (100)	–	–	–
Total	464	133 (29)	12 (9)	40 (30)	28 (21)	38 (29)	15 (11)

*–, absence of genotypes. †Born before January 1, 2006, or <4 months of age. ‡Children were considered vaccinated if they had received 2 doses of vaccine. §All stool samples were negative for rotavirus.

In the 18 months from July 2006 through December 2007, almost all RV-A–positive samples (35/36, 97%) showed G2P[4] specificity, which suggests a shift in genotype distribution, characterized by an increase in G2P[4] detection since 2006. When the pre- and postvaccination periods were compared, these changes in genotype distribution were found to be accompanied by a significant reduction in the detection rate of RV-A from 38% (73/193) in 2005 to 24% (26/109) in 2007 (p = 0.012 by χ^2^ test). Vaccination rates in the RV-A–positive and –negative groups (considering only children eligible for full vaccination) were 29% (4/14) and 58% (35/60), respectively (odds ratio 0.29; 95% confidence interval 0.07–1.15; p = 0.043 by Fisher exact test, 1-sided. The 4 RV-A–positive vaccinated children were infected with G2P[4] genotype.

## Conclusions

The first studies that assessed the RV-A genotype distribution after the introduction of Rotarix were carried out in northeastern Brazil (*3*–*5*). They offered the hypothesis that vaccination with the monovalent G1P[8] vaccine possibly created conditions in which RV-A G2P[4] could acquire selective advantage over P[8] genotypes (*5*). Nevertheless, a temporal periodicity, within the ≈10-year cyclic pattern of G2P[4] occurrence in Brazil, should be considered to explain the increased detection of this genotype since 2006. This periodicity could coincide with RV-A vaccine introduction and the consequent reduction of circulation of non-G2 strains.

G2P[4] RV-A was not detected from 2000 to 2004 in Rio de Janeiro (*2*–*6*); it was identified in 2005 (1.4%) and reemerged in 2006 (41%). Similarly, in northern Brazil, RV-A G2P[4] was detected in 2005 after a period of absence (A. Linhares, pers. comm.). When other Latin American countries are considered, an outbreak of RV-A gastroenteritis with a high rate of G2P[4] detection was recently described in Honduras (*12*). According to Patel et al. (*13*), ongoing surveillance in El Salvador, Guatemala, and Honduras showed that G2P[4] was the predominant circulating strain in 2006 (68%–81%). In Argentina, this genotype was also circulating in 2006 (J. Stupka, pers. comm.). RV-A with short electropherotype, characterized as G2P[4], was detected at high frequency in 2005 in Paraguay, after a 6-year absence (*14*). In these South American countries that border Brazil, there are no RV-A immunization campaigns, and G2P[4] was detected before introduction of Rotarix in Brazil.

In Bangladesh, the rate of G2P[4] detection increased recently (43% in 2005–2006) (*15*). The detection of G2P[4] since 2005 in countries where RV-A vaccination is not implemented reinforces the possibility of natural reemergence of this genotype.

Our data also suggest a significant reduction in the rate of RV-A detection between the pre- and postvaccination periods. The comparison of vaccination rates between RV-A–positive and –negative children, even with a small sample size, suggests that vaccinated children have a reduced risk for severe RV-A diarrhea.

This survey is among the first to evaluate the effects of Rotarix in Brazil, the first Latin American country to introduce universal rotavirus vaccination. We believe that the emergence of strains that may escape protection can be a challenge to the RV-A immunization program in Brazil and needs to be continuously monitored.
